# Comment on Experimental Determination of the Threshold Dose for Bifidogenic Activity of Dietary 1-Kestose in Rats. *Foods* 2020, *9*, 4

**DOI:** 10.3390/foods9040519

**Published:** 2020-04-21

**Authors:** Yihao Shen, Yang Shi, Zhongke Sun

**Affiliations:** 1 Institute of Food and Drug Inspection, Zhoukou Normal University, Zhoukou 466001, China; 2 College of Chemistry and Molecular Engineering, Zhengzhou University, Zhengzhou 450001, China

**Keywords:** prebiotics, bifidogenic activity, Bifidobacterium, fructo-oligosaccharide (FOSs) dose, quantitative real-time polymerase chain reaction (qPCR)

Currently, our group is undertaking a program trying to evaluate the bifidogenic effect/activity of different prebiotics and their dose-effect relationships. Therefore, we were interested to read an article reported the determination of the minimal threshold dose for the bifidogenic activity of dietary 1-kestose in rats published in *Foods* in December 2019 [1]. It was reported that *Bifidobacterium* spp. preferentially metabolized fructo-oligosaccharides (FOSs) with a lower degree of polymerization [2]. As the major component of FOSs with the smallest molecular weight, determination of its minimal threshold dose for the bifidogenic activity of 1-kestose in foods is definitely important. However, we have some concerns, especially on the methodological drawbacks, which may be critical to this report.

Watanabe et al. measured cecal microbiota populations using quantitative real-time polymerase chain reaction (qPCR) technology [1]. The 16S rRNA was amplified as a target gene for quantification of *Bifidobacterium* spp. using a pair of primers (F: GATTCTGGCTCAGGATGAACGC; R: CTGATAGGACGCGACCCCAT). The authors provided a reference (Reference 8) for the primers. However, we noticed that paper enumerated *Bifidobacterium* spp. by traditional cell counting on agar plate other than qPCR amplification [3]. It is obvious the citation is incorrect.

Then, we found the above primers in another paper, which came from the same research group, in which a similar experiment was conducted investigating the effect of 1-kestose within a range of 0.5–5.0% [4]. In this paper, the primers have the same sequences but different names (Bif LM26F/Bif 228R). Although it was primarily designed based only on the corresponding gene of *Bifidobacterium longum* subsp. *longum* JCM 1217^T^ (*B. longum*), this pair of primers was used as the *Bifidobacterium* genus specific primer. In fact, the primers (Bif LM26F/Bif 228R) were first designed in 2004 [5]. The applicability/university of the primers was evaluated with 10 type strains of *Bifidobacterium* spp., representing 10 different species. The specificity of the primers was checked with 21 type strains of nonbfidobacerial strains. Thereafter, the primers were used in many studies. However, we now know there are more than 88 species in the genus of *Bifidobacterium* and the diversity of gut microbiota is quite high [6]. Therefore, we wonder whether the primers are reliable for genus-specific amplification.

To support our speculation, we performed a specificity check of this pair of primers using Primer-BLAST with default settings [7]. Results produced online indicate both the specificity and universality of this pair of primers are questionable. According to this prediction, the primers yield ~230 bp amplicons from different genera of bacteria, targeting the 16S rRNA gene. It was equally efficient for *Gardnerella* spp., *Scardovia inopinata*, and *Parascardovia denticolens*, albeit could amplify many species of *Bifidobacterium* spp. Unfortunately, representatives of these bacteria were not included in the original study [5]. Efficient detection of many species of *Bifidobacterium* could only be possible with one mismatch in primers. With two mismatches in primers, even *Bacillus megaterium, Chryseobacterium indologenes, Synergistales* bacterium, and *Microbacterium* spp. could be amplified as easily as *B. animalis*. Moreover, many strains belonging to *B. saguini, B. callitrichos, B. mongoliense, B. psychraerophilum, B. catenulatum, B. pseudolongum, B. reuteri,* and *B. longum* could only be detected with three mismatches in primers. At the same time, more than 12 other genera of bacteria could be positively amplified under such conditions. Details of representative untargeted microbes that could be potentially detected with this pair of primers were summarized (Table 1). Therefore, it seems this pair of primers is neither universal nor specific to *Bifidobacterium* spp.

Thirdly, *B. pseudolongum* was recently identified as almost the sole bifidobacterial species (>95%) after FOS (containing 60.6% 1-kestose) treatment in mice by a comprehensive metagenomic study [8]. Further study indicated that FOS could selectively promote *B. pseudolongum* proliferation in both the lumen and the mucosa from the cecum to the distal colon in mice [9]. If this is also the case in rats, results obtained with primers having as many as three mismatches, as predicted by Primer-BLAST, may not be reliable.

Fourthly, details of the qPCR analyses were not described in the cited reference by the authors [4], but in another reference [10]. From that paper, we know the authors used 20 ng genomic DNA as a template and SYBR green as fluorescent dye during qPCR, and included melting curves. However, no data were supplied about Ct values and melting temperatures; these data are important for quality evaluation and reproducibility. From the normalized data demonstrated in Figure 1, it seems the qPCR data were of low quality, as indicated by the large standard error of mean (SEM). Meanwhile, information on repeats was unavailable.

Lastly, but importantly, the authors concluded that the minimum dose of dietary 1-kestose to induce significant bifidogenic activity in rats was 0.3% by weight in the diet. In contrast, Tandon et al. reported that intake of FOS appears to result in a statistically significant increase in the abundance of most of the operational taxonomic units (OTUs) belonging to *Bifidobacterium* in humans, irrespective of amount; even as low as 2.5 g/d was administered [11]. In fact, we observed significant stimulation of bifidobacteria using as low as 0.125–0.25% FOS and other prebiotics during in vitro fermentation, in our unpublished study. Meanwhile, the authors claimed that 0.16 g/d/kg of rat body weight (0.3% 1-kestose in feed) was roughly equivalent to 10 g/d (~0.16 × 60) for humans of around 60 kg body weight. We wonder whether such a calculation is appropriate, as the sensitivity to a reagent in rats and humans is often different. In toxicological studies, the sensitivity index between humans and rats is 6.25 in common [12]. Therefore, we call more attention to the method and conclusion. More studies are required to determine the minimum effective dose of 1-kestose in rats and feeding studies in humans are needed before making a recommendation as a functional food.

## Figures and Tables

**Table 1:** Primer specificity analysis using Primer-BLAST.

Number of Mismatch	Untargeted Microbes	Amplicon Size (bp)
**0**	*Gardnerella* spp.	231
	*Scardovia inopinata*	235
	*Parascardovia denticolens*	232
**1**	Uncultured bacterium	230–235
**2**	Uncultured bacterium	227–235
	*Synergistales* bacterium	229
	*Microbacterium* spp.	230
	*Chryseobacterium indologenes*	160
	*Bacillus megaterium*	189
**3**	*Slackia* spp.	231
	*Raoultibacter* spp.	230
	*Microbacterium* spp.	226–232
	*Arabia massiliensis*	230
	*Nesterenkonia* spp.	234
	*Cellulomonas* spp.	228–231
	*Actinomyces* spp.	238
	*Enorma* spp.	227
	*Collinsella* spp.	227
	*Arthrobacter* spp.	232
	*Lysinimonas* spp.	230–232
	*Eggerthellaceae* bacterium	232
